# The X-Ray in the Treatment of Malignancy

**Published:** 1915-07

**Authors:** B. T. Van Zant

**Affiliations:** Houston, Texas


### The X-Ray in the Treatment of Malignancy.

BY B. T. VAN ZANT, M. D., HOUSTON, TEXAS.

Of all therapeutic measures aimed at amelioration or cure of malignant conditions in recent years, radiant energy, in its various forms, easily takes first place. No other agency yet tried has offered so much in the way of results. Potent for good when properly used, yet fraught with greatest danger improperly or unskilfully used.

The application of this radiant energy is so recent, its action so little understood and the statements of investigators so conflicting it is small wonder the laity and even the medical profession are willing to ascribe to it magic powers and selective specific action. Therapeutically, chemically, physically and physiologically all forms of radiant energy are so nearly identical that, while my remarks are addressed especially to X-ray administration, they apply in a measure to all. The real difference lying only in the difference in amount and penetration powers.

For a proper understanding of the therapeutic application of X-rays, it is necessary that we have a clear understanding of their nature and their production.

X-rays are generated by the bombardment of a metal surface usually platinum or tungsten in a more or less perfect vacuum by a stream of cathode rays. The cathode rays are generated by an electric current, and their velocity depends upon the voltage of the generating current; in other words, upon the pressure or stress of the current—less the resistance encountered in the partial vacuum—penetrated. The amount of rays depends upon the ameperage or amount of the generating current.

Now comparing this cathode stream to a jet of water under high pressure flowing from a nozzle—the higher the pressure, the greater the velocity—the larger the nozzle, the greater the volume. The center of this stream overcomes forward resistance and moves rapidly, while the surrounding portions, layer by layer, become more and more retarded by atmospheric friction, until the outermost layer looses velocity and is dissipated. Now should this stream impinge upon a hard surface, the central portion rebounds to a great distance with great velocity, while the more slowly moving outer layers strike with less force and rebound to lesser distance. Thus we have in the X-ray tube when excited a reflection of rays of enormously different velocities; some extremely high, hard and penetrating in proportion to the voltage; also all gradations of weaker and weaker rays until those of least velocity are so weak, or, as we call them, soft, they strike the wall of the tube without penerating it, thus giving rise to its characteristic fluorescence. Other rays barely penetrate the walls of the tube, only to be absorbed by the first layers of atmosphere encountered. For this reason, the volume of X-rays diminishes rather more than as the square of the distance from the tube.

Up to the present time, no thoroughly satisfactory method of determining either the amount or the penetration of the rays has been found, and no practical instruments devised. Yet the greatest importance attaches to the selection of the proper penetration and the amount administered in any given case.

Under the circumstances, then, it is small wonder that the use of the rays has been haphazard.

Thousands of physicians have applied the rays in tens of thousands of cases, with no conception whatever of the nature action, or danger of the agency employed. Consequently, results have varied; many cases have been brilliantly cured; others materially benefited, while probably a still larger number have proven negative or been made much worse, due to a stimulating action of insufficient raying.

If some cells of the body—mesoderm, ectoderm or intoderm—get the habit of subdividing, and the products of this subdivision fail to reach the adult type, you have a case of malignancy to deal with. We have not space to consider the varieties and subvarieties, hence we apply to the term malignant in its very broadest meaning.

Surface malignancy, such as the breaking down "hyperkeratotic patch or the ulceration of a "mole," yields promptly and permanently to rays applied to medium penetration and just sufficient to produce a moderate erythema. They should be treated without filter and at 8 to 10 inches from the tube.

Deep therapeutic raying, to be effective, requires finished technique and a thorough understanding of the apparatus, filters, skin reaction and deep tissue reaction. Enough rays applied means a destruction of the malignant cells to a certain extent, but the real curative effect is due to the fact that certain changes are brought about in the cells just about to undergo division whereby the division is inhibited and the younger cells permitted to grow almost to the adult type, where their further power to reproduce is destroyed. For this reason, I believe it is wise to apply the rays from time to time in moderate amounts, rather than as has been done by some operators—apply an overwhelming dose at one sitting. The analogue of this inhibition of cell division is found in the a-zoospermia produced by raying the tests, whereby cell division of the spermatoblastic cells is entirely stopped.

Tissues absorb large quantities of rays, and only recently have generators and tubes been perfected to the point where sufficiently abundant, very penetrating rays could be produced, without an objectionable amount of the soft rays, to make deep raying effective.

Filtration through aluminum, 1 to 5 mm., using thin leather under the aluminum to protect the skin from the specific soft secondary aluminum rays, removes large amounts of the soft rays. Greater distance of the tube from the part allows the application of more homogeneous rays, and the system of cross-fire administration render deep raying not only possible but practical.

In deep raying the surface is mapped out in equal small areas and marked off with silver nitrate; the entire field is protected with lead or by treatment speculac, except the area to be rayed; the tube is adjusted so that the line of central rays penetrate directly the disease tissues—super hardened tube—highest inductance and the rays poured in equal to an erythema dose for that area of skin exposed. Another area is next exposed in the same manner until all the divisions have been exposed. Thus, while no portion of the skin has been overexposed, the cross-fire has applied enormous amounts of rays to the deeper tissues. Judgment must be used, or excessive raying of the tumor mass may produce excessive destruction; absorption becomes inadequate and elimination overcrowded, whereby a toxic effect is produced in the whole system. Much evidence goes to indicate that the destruction of a malignant mass in amounts in excess of the ability of the economy to eliminate not only produce toxemia but through inhibition of the lymphatic system lessens phagocytosis and produces a condition peculiarly susceptible to the advance of the malignant process.

The problem of treatment becomes then a matter of understanding of the kind and amount of tissue involvement, the kind and amount of rays to be administered; not a 42 c.m. gun to demolish a mole hill, nor an air rifle to sink a dreadnaught. To the question—when, where and wherefore let us answer—know —then, therefore,—make all cases beneficial by knowing the kind, the amount and the way to apply the rays.

